# Maternal Undernutrition Modulates Neonatal Rat Cerebrovascular Structure, Function, and Vulnerability to Mild Hypoxic-Ischemic Injury via Corticosteroid-Dependent and -Independent Mechanisms

**DOI:** 10.3390/ijms22020680

**Published:** 2021-01-12

**Authors:** Patsy Naomi Franco, Lara M. Durrant, Coleen Doan, Desirelys Carreon, Alejandra Beltran, Amandine Jullienne, Andre Obenaus, William J. Pearce

**Affiliations:** 1 Center for Perinatal Biology, School of Medicine, Loma Linda University, Loma Linda, CA 92350, USA; nafranco@llu.edu (P.N.F.); lara.durrant@gmail.com (L.M.D.); cdoan@llu.edu (C.D.); decarreon@llu.edu (D.C.); albeltr@bu.edu (A.B.); 2 Institute for Memory Impairments and Neurological Disorders, University of California, Irvine, CA 92697, USA; ajullien@uci.edu; 3 Department of Pediatrics, University of California, Irvine, CA 92697, USA; obenausa@uci.edu; 4 Preclinical and Translational Imaging Center, University of California, Irvine, CA 92697, USA

**Keywords:** cerebral arteries, fetal programming, myogenic reactivity, myosin, smooth muscle phenotype

## Abstract

The present study explored the hypothesis that an adverse intrauterine environment caused by maternal undernutrition (MUN) acted through corticosteroid-dependent and -independent mechanisms to program lasting functional changes in the neonatal cerebrovasculature and vulnerability to mild hypoxic-ischemic (HI) injury. From day 10 of gestation until term, MUN and MUN-metyrapone (MUN-MET) group rats consumed a diet restricted to 50% of calories consumed by a pair-fed control; and on gestational day 11 through term, MUN-MET groups received drinking water containing MET (0.5 mg/mL), a corticosteroid synthesis inhibitor. P9/P10 pups underwent unilateral carotid ligation followed 24 h later by 1.5 h exposure to 8% oxygen (HI treatment). An ELISA quantified MUN-, MET-, and HI-induced changes in circulating levels of corticosterone. In P11/P12 pups, MUN programming promoted contractile differentiation in cerebrovascular smooth muscle as determined by confocal microscopy, modulated calcium-dependent contractility as revealed by cerebral artery myography, enhanced vasogenic edema formation as indicated by T2 MRI, and worsened neurobehavior MUN unmasked HI-induced improvements in open-field locomotion and in edema resolution, alterations in calcium-dependent contractility and promotion of contractile differentiation. Overall, MUN imposed multiple interdependent effects on cerebrovascular smooth muscle differentiation, contractility, edema formation, flow-metabolism coupling and neurobehavior through pathways that both required, and were independent of, gestational corticosteroids. In light of growing global patterns of food insecurity, the present study emphasizes that infants born from undernourished mothers may experience greater risk for developing neonatal cerebral edema and sensorimotor impairments possibly through programmed changes in neonatal cerebrovascular function.

## 1. Introduction

The US Department of Agriculture estimates that in 2019, at least 870 million people worldwide regularly experienced food insecurity, including 35 million in the United States [1], and these numbers likely will increase due to the COVID-19 pandemic [2]. This food insecurity, and the nutritional insufficiency it causes, can lead to numerous detrimental physiological consequences, particularly in pregnant mothers and their offspring [3,4]. Frequently, undernourished mothers give birth to infants that initially appear healthy but develop multiple metabolic and cardiovascular disease profiles in middle age, as first reported by David Barker [5]. Correspondingly, numerous studies now recognize “Developmental Origins of Health and Disease” as the concept that an adverse intrauterine environment can cause the delayed appearance of adult-onset cardiovascular and metabolic disease [6,7].

Investigation of the mechanisms that mediate “Developmental Origins of Health and Disease” have led to the hypothesis of “Fetal Programming”, which posits that fetal stressors precipitate structural, metabolic, and genetic changes that persist through adulthood [8,9]. In turn, the stressors that drive such changes include not just poor maternal nutrition and hydration, but also environmental challenges (e.g., hypoxia, hyperthermia, pollution) [10,11,12], compromised maternal health (organ disease, infections, drug abuse) [13,14,15], and placental disease (abruption, accreta, previa, ischemia, etc.) [16]. The consequences of fetal programming typically influence cardiovascular, renal, and endocrine development, which together increase risks for adult onset obesity, glucose intolerance, hypertension, coronary insufficiency, and renal dysfunction [4,17]. Fetal programming also affects the brain and cerebrovascular circulation resulting in altered brain structure with increased risk for learning disabilities and cognitive dysfunction [4,18]. In addition, fetal programming also appears to increase the risk for cerebrovascular disease in offspring from food-restricted mothers [3,19].

Early investigations into the mechanisms governing fetal programming sought to explain the phenomenon of “catchup growth”, in which nutrient-deprived fetuses gained weight more rapidly than normal as neonates, when given access to abundant food [6]. Initial morphological studies focused on the hypothesis that nutrient restriction might attenuate fetal arteriogenesis [20,21] and renal tubule formation [22], thus leading to lasting changes in postnatal cardiovascular/renal structure, function and health [17]. More recent studies of fetal programming, however, have implicated regulatory changes in multiple key genes, which in turn, help explain persistent changes in postnatal cardiovascular structure and function [6,9]. These regulatory epigenetic changes include methylation of cytosine residues of DNA, particularly in gene promoter regions, chemical modifications of the histone proteins that govern DNA supercoiling and gene silencing, and production of miRNA sequences, many of which originate from intronic regions of freshly transcribed mRNA [6,23]. Given their essential roles as mediators of stress, endocrine mechanisms constitute another important component of fetal programming, and among these the key signaling molecules of the hypothalamic pituitary adrenal (HPA) axis, including corticotropin releasing factor, adrenocorticotrophic hormone (ACTH) and corticosteroids, have attracted considerable interest [24].

As the main effectors of the HPA axis, corticosteroids bind to ubiquitously-expressed glucocorticoid and mineralocorticoid receptors [25,26], which during key developmental periods affect patterns of gene transcription that govern the structural and functional maturation of multiple tissues and organs [24]. Correspondingly, the levels of corticosteroids during pregnancy are critical, as excess glucocorticoids, either endogenous or synthetic, can inhibit multiple embryonic processes including angiogenesis [27] and placental production of corticotropin releasing hormone [8]. Not surprisingly, the transfer of maternal corticosteroids to the fetus is highly regulated; placental deficiency of the enzyme 11-beta-hydroxysteroid dehydrogenase type 2, which metabolizes and inactivates cortisol and corticosterone and can be depressed by maternal undernutrition [26], is associated with growth restriction, malformations and other developmental complications [8]. Tools to study the function of corticosteroids during pregnancy include specific inhibitors of 11-β hydroxylase and aldosterone synthase activity, such as metyrapone (MET), a reversible corticosteroid synthesis inhibitor [28,29]. In animal experiments, MET has proven useful in studies of the long-term programming effects of excess corticosteroid exposure in utero [30,31], and in clinical settings, MET can help diagnose and treat adrenal insufficiency [29].

Previous studies of fetal programming have identified a significant impact on vascular smooth muscle and its development [31,32]. Specifically, these studies have demonstrated that the phenotype of vascular smooth muscle, as indicated by differential expression of contractile proteins such as smooth muscle αactin and myosin heavy chain isoforms [33,34], can be potently modulated by fetal stress [35,36]. Correspondingly, changes in smooth muscle phenotype in response to the cardiovascular stresses imposed by hypoxia and ischemia, can alter both resting myogenic vascular tone and contractile responses induced by depolarizing stimuli, contractile agonists, and vasodilators [37,38]. A majority of studies of how fetal programming influences postnatal vascular structure and function have focused on mesenteric, renal and femoral arteries and have revealed attenuation of angiogenesis [27], reduced contractile responses to phenylephrine and norepinephrine [39], and reduced vasodilatory responses to acetylcholine, bradykinin, sodium nitroprusside, and presumably nitric oxide [40]. Although relatively few studies have explored the effects of fetal programming on the contractility of neonatal cerebral arteries, the findings reported to date reinforce the idea that fetal programming potently modulates neonatal cerebrovascular reactivity [35,36]. These studies also imply that corticosteroids contribute significantly to the effects of fetal programming on cerebrovascular development and functional maturation.

In light of the relative paucity of studies of the cerebrovascular consequences of fetal programming, the present study explores the hypothesis that an adverse intrauterine environment caused by maternal undernutrition (MUN) during the latter half of gestation acts through corticosteroid-dependent and -independent mechanisms to program lasting functional changes in the neonatal cerebrovasculature. The model employed imposed a long-term fetal stress via 50% restriction of total caloric intake as defined by an ad libitum pair-fed control pregnant dam, during the last 11 days of gestation, as previously described [31,36]. Also as previously described [35,36], our model included administration of MET at 0.5 mg/mL in the drinking water during the last 10 days of gestation to attenuate circulating levels of maternal and fetal corticosteroids. As a short-term cardiovascular stress, our model also included a mild hypoxic-ischemic challenge induced by unilateral carotid ligation at P9/P10 followed 24 h later by 90 min of exposure to 8% O_2_ and harvest at P11/P12. The mild hypoxic-ischemic (HI) insult used in our model generally produced no large infarcts, which rendered it optimally relevant to the mild patterns of injury characteristic of the majority of HI brain injuries in human neonates [41]. The endpoints measured included circulating levels of corticosterone to track involvement of the HPA axis, changes in the smooth muscle phenotype of cerebral arteries as indicated by confocal microscopy, and the contractility of neonatal cerebral arteries as revealed by myography. In addition, pups underwent ex vivo MRI to assess T2 relaxation times as an index of blood brain barrier function and edema formation, and apparent diffusion coefficients (ADC) calculations to estimate changes in mean water diffusivity as a measure of cellular swelling and health. At the level of the whole organism, the experimental design also included assessments of neurobehavior to reflect the integrated effects of all perturbations. Together, these experiments combined cellular, tissue, organ, and organismal endpoints to provide a unique view of how the long-term stress of MUN interacted with the short-term stress of hypoxic ischemia to modulate both corticosteroid-dependent and -independent influences on the neonatal brain and its vasculature.

## 2. Results

### 2.1. General Findings

**Figure 1:**
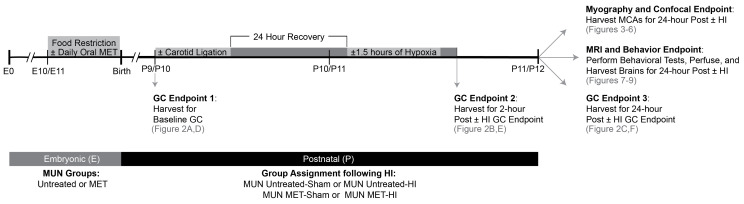
Experimental Overview of Embryonic and Postnatal Events. As indicated in the above diagram (not to scale), all pregnant Sprague–Dawley rats received a diet of 50% caloric reduction beginning at E10 and continuing until term, resulting in maternal undernutrition (MUN). Half of the animals also received a daily oral dose of 0.5 mg/mL of metyrapone (MET) in the drinking water beginning at E11 through term, yielding two groups: the Untreated group (untreated water), and the MET group (MET-treated water). After birth, the euthanization of pups at two specific endpoints (GC Endpoints 1 and 2) followed by trunk blood collection allowed for the determination of plasma glucocorticoid (GC) levels before and immediately following hypoxic-ischemic (HI) injury. Plasma collection for the third GC Endpoint, artery harvesting for myography and future confocal measurements, and behavioral testing followed by perfusion fixation for future ex vivo MRI evaluation occurred in cohorts of P11/P12 rats 24 h following HI injury.

These protocols used a total of 244 MUN neonates from 30 litters, that provided 78 arteries from 17 of the litters for the following endpoints (1) corticosterone time course; (2) confocal and myography; and (3) MRI and behavior. Each pup brain could provide up to four artery segments; however, not all endpoints required isolated arteries. The experimental endpoints included two prenatal groups: a MUN Untreated group with 137 neonates from 14 litters and a MUN MET-treated group with 107 neonates from 16 litters (Figure 1). Additionally, the 78 neonatal arteries included 34 untreated arteries from eight litters and 44 MET-treated arteries from nine litters.

On average, each rat mother gave birth to 13 pups from which urogenital distances determined sex. Paired pups of each sex from every litter underwent either Sham surgery or HI surgery for all experiments. ANOVA revealed no sexual dimorphism for any of the endpoints, consistent with other studies of the neonatal cerebrovasculature [42] including our own [35]. Statistical analysis relied on pooled groups of males and females.

At birth, MUN pups weighed less (5.2 ± 0.2, *n* = 137)) when compared to Control Diet (CD) pups (6.6 ± 0.1 g, *n* = 144), which demonstrated that this model of MUN induced statistically significant intrauterine growth restriction in the rat. The presence of MET in MUN dams did not affect these decreases in pup birth weights (5.2 ± 0.2 g, *n* = 88). At P11/P12, MUN Untreated-Sham pups (18.3 ± 0.5 g, *n* = 42) weighed significantly less than CD Untreated-Shams (19.3 ± 0.5 g, *n* = 13). Further, at this age, the MUN Untreated-Sham pups weighed significantly less than MUN MET-Sham pups (20.3 ± 0.2 g, *n* = 32). At 24 h following the hypoxic insult, the weights of MUN Untreated-HI pups and MUN Untreated-Sham pups did not differ. Gestational MET, however, unmasked a significant difference between the MUN MET-Sham (20.3 ± 0.2 g, *n* = 32) and MUN MET-HI pups (18.3 ± 0.4 g, *n* = 34).

### 2.2. Blood Collection and Corticosterone Analysis

**Figure 2:**
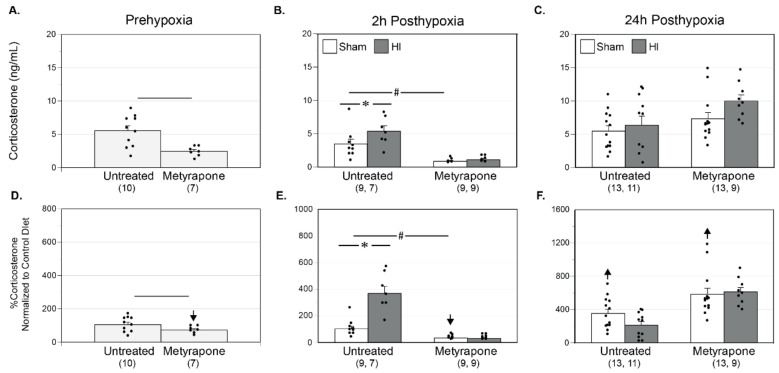
Effects of HI, gestational MET and MUN on the time course of plasma corticosterone. Plasma corticosterone measurements occurred at the time of sacrifice in the four treatment groups at three time points in MUN pups. (**A–C**) depict the effects of MET programming and mild HI on the raw responses of MUN groups. (**D–F**) The isolated effects of MUN alone, obtained by normalizing the raw response to the corresponding responses observed in Control Diet (CD) animals. (**A**,**D**) Corticosterone concentrations measurements took place immediately before surgery as pre-treatment baseline. (**B**,**E**) Assessments of corticosterone levels happened 2 h post-hypoxia or normoxia in ligated or Sham-operated animals 24 h prior, respectively. (**C**,**F**) Analysis of plasma corticosterone also ensued 24 h post-hypoxia or normoxia in Ligated or Sham-operated pups. (**A–C**) Columns and error bars overlaid with dot plots indicate SEM. ANOVA with a post-hoc Fisher’s LSD determined the statistical significance as shown by: solid horizontal bars for significant differences between Untreated and MET baseline groups, * for differences between Sham and HI groups and # for differences between MET-Sham and Untreated-Sham groups. Due to unequal variance for groups portrayed in Figure 2B, an independent *t*-test determined the significance between Untreated-Sham and HI groups. (**D–F**) Columns and error bars overlaid with dot plots indicate MUN plasma corticosterone values normalized to CD values for each time point. Up and down arrows denote significant differences (*p* < 0.05) between normalized values of MUN and 100% for both Untreated- and MET-Sham groups; an up arrow indicates a significant enhancement by MUN whereas a down arrow indicates a significant attenuation by MUN. To improve visual clarity, significant differences between Untreated-HI and MET-HI are not shown. Parentheses at the bottom of each graph display *n* values for each group.

The ELISA assay used in these studies could detect a difference of 20.9% with a power of ≥0.8 for the number of pups used (*n* = 97). For comparisons of raw baseline plasma corticosterone (GC) measurements between MUN groups (see Figure 1, GC Endpoint 1), gestational MET transformation significantly reduced GC levels by approximately 2-fold when compared to the MUN Untreated group (Untreated: 5.5 ± 0.8 ng/mL; MET: 2.5 ± 0.3 ng/mL) (Figure 2A). At two hours following the hypoxic insult (see Figure 1, GC Endpoint 2), HI significantly elevated GC levels relative to the MUN Untreated-Sham group (Sham: 3.5 ± 0.7 ng/mL; HI: 5.4 ± 0.8 ng/mL); gestational MET treatment ablated this effect (Figure 2B). Additionally, MET transformation significantly depressed levels of corticosterone when compared to both MUN Untreated-Sham (MET: 0.8 ± 0.1 ng/mL) and MUN Untreated-HI (MET: 1.1 ± 0.2 ng/mL). For the final measurement at 24 h post-hypoxia (see Figure 1, GC Endpoint 3), no effect of HI existed between MUN Untreated-Sham (5.5 ± 0.8 ng/mL) and MUN Untreated-HI (6.4 ± 1.3 ng/mL) or between MUN MET-Sham (7.3 ± 0.9 ng/mL) and MUN MET-HI (10.0 ± 0.9 ng/mL) (Figure 2C).

When comparing the effect of the MUN diet on GC levels relative to those in CD pups, normalization revealed that GCs levels were significantly less at baseline in normalized Untreated pups (Figure 2D). Moreover, for between-group comparisons of MUN plasma values normalized to CD, plasma corticosterone averaged to values significantly less in MET pups than in Untreated pups. At 2 h post-hypoxia, relative to CD values, normalized plasma corticosterone values were significantly greater in Untreated-HI pups (Figure 2E). Acute hypoxia resulted in significantly greater GC levels in the normalized Untreated-HI group relative to the normalized Untreated-Sham group. MET ablated this effect. Relative to CD values, MUN values in MET-Sham and MUN MET-HI pups were significantly less at this timepoint. Additionally, normalized values of plasma corticosterone in MET-Sham and MET-HI were significantly less relative to normalized values in Untreated-Sham and Untreated-HI groups, respectively. At 24 h post-hypoxia, relative to the CD groups, normalized values of plasma corticosterone were significantly greater in all groups, and normalized GC values were significantly greater in the MET-HI group relative to the Untreated-HI group (Figure 2F). In summary, these results demonstrated the robust programming effects of MUN indicated by enhanced HPA reactivity to acute levels of HI injury, depressed adrenal reactivity in MET-transformed pre-hypoxic and post-hypoxic (acute only) pups, and generally augmented levels of GC in Sham and HI pups 24 h post-hypoxia.

### 2.3. Confocal Microscopy

**Figure 3:**
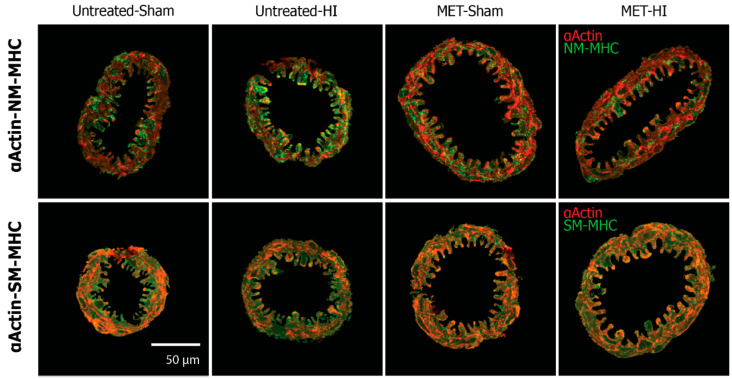
Effects of HI and gestational MET on Confocal Colocalization of Smooth Muscle Contractile Proteins in MUN Arteries. The images shown originated from middle cerebral arteries stained for smooth muscle αactin (αActin) and non-muscle myosin heavy chain (NM-MHC) on the **top panel** or αactin and smooth muscle MHC (SM-MHC) on the **bottom panel** from each of the four MUN treatment groups.

**Figure 4:**
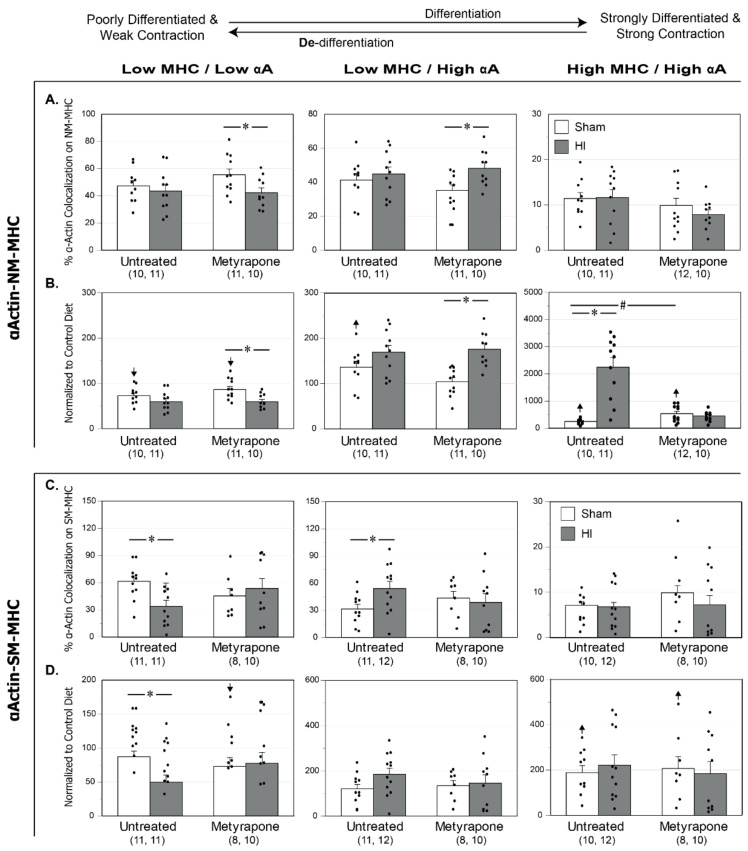
Effects of HI, gestational MET and MUN on Confocal Colocalization of Smooth Muscle Contractile Proteins. Confocal microcopy allowed for the assessment of contractile protein organization of αActin with two different myosin heavy chain isoforms: (a) NM-MHC, and (b) SM-MHC. (**A**,**B**) depict NM-MHC data, while (**C**,**D**) depict SM-MHC data. Confocal coefficients of colocalization were sorted into three distribution groups: Low MHC/Low αActin, Low MHC/High αActin, High MHC/High αActin. For (**A**,**C**), within each experimental group, all subgroups totaled to 100%. Note that the vertical scale for each of the three distribution groups differ (100% vs. 80% vs. 30%). In relation to smooth muscle phenotype, a leftward shift in pixel counts (e.g., decreased High-High and increased Low-Low values) suggests contractile de-differentiation, and vice versa. Columns and error bars overlaid with dot plots indicate SEM for raw MUN data. For (**B**,**D**) columns and error bars overlaid with dot plots indicate MUN colocalization values normalized to CD values for each category. ANOVA with a post-hoc Fisher’s LSD determined the statistical significance as shown by: * for differences between Sham and HI groups and # for differences between MET-Sham and Untreated-Sham groups. Up and down arrows denote significant differences (*p* < 0.05) between normalized values of MUN and 100% for both Untreated- and Metyrapone-Sham groups; an up arrow indicates a significant enhancement by MUN whereas a down arrow indicates a significant attenuation by MUN. To improve visual clarity, significant differences between Untreated-HI and MET-HI are not shown. Parentheses at the bottom of each graph display *n* values for each group.

Confocal microcopy allowed for the assessment of contractile protein organization of smooth muscle αactin (αActin) with two different myosin heavy chain isoforms: (a) non-muscle myosin heavy chain (NM-MHC), and (b) smooth muscle myosin heavy chain (SM-MHC) (Figure 3 and Figure 4). Figure 3 depicts the confocal images for each group, while Figure 4 portrays the percentage pixel distributions among the Low-Low, Low-High, High-High phenotype categories for each experimental group. A key characteristic of these pixel distributions is that the percentages in each of the three categories total to 100% for each experimental group. Expression of NM-MHC decreases with vascular maturation [32,34]. SM-MHC becomes the predominant isoform and reflects a more mature, contractile-efficient phenotype with approximately twice the contractile efficiency as NM-MHC [43]. In relation to smooth muscle phenotype, a rightward shift in pixel distributions (e.g., decreased Low-Low and increased Low-High values) for colocalization of αActin with MHC, suggested contractile differentiation. The methods used to measure αActin -NM-MHC colocalization values could detect differences of 12.8% (Low-Low), 12.5% (Low-High), and 20.2% (High-High) with a power of ≥0.8 for the number of pups in each quadrant (*n* = 43). In arteries from pups whose dams received MET, mild HI reduced colocalization in the Low-Low category and increased colocalization in the Low-High category, revealing a rightward shift in pixel distributions and contractile differentiation in moderately differentiated smooth muscle (Figure 4A).

Relative to the CD values of colocalization, MUN colocalization values in the normalized Untreated-Sham group were significantly less in the Low-Low category and significantly greater in both the Low-High and High-High categories (Figure 4B). These changes revealed a significant rightward shift in pixel distributions that implied contractile differentiation. Additionally, normalized Untreated-HI and normalized MET-HI groups showed the same pattern of changes as the normalized Untreated-Sham, indicating contractile differentiation. The normalized MUN MET-Sham groups displayed normalized values of colocalization that were significantly less in the Low-Low category, and significantly greater in the High-High category, which also revealed a rightward shift of pixel distributions.

Similar to changes between the raw MUN group results (Figure 4A), analysis of changes between the normalized groups revealed that colocalization values were significantly less in the Low-Low category and significantly greater in the Low-High category in the MET-HI groups than for values in the MET-Sham group (Figure 4B). This rightward shift in pixel distributions implied contractile differentiation in moderately differentiated smooth muscle. Additionally, in the High-High category, normalization unmasked significantly greater colocalization values following mild HI in the Untreated groups. In this same category (High-High), normalization also revealed that colocalization values were significantly greater in MET-Sham than in Untreated-Sham arteries, but these values were significantly less in MET-HI than in Untreated-HI arteries.

The methods used to measure αActin-SM-MHC colocalization values could detect differences of 23.8% (Low-Low), 25.9% (Low-High), and 33.5% (High-High) with a power of ≥0.8 for the number of pups in each quadrant (*n* = 41). For effects between MUN groups (Figure 4C), mild HI resulted in a significant decrease in colocalization values in the MUN Untreated-HI arteries relative to MUN Untreated-Sham arteries in the Low-Low category and an increase in the Low-High category, revealing a right shift in pixel distributions consistent with a pattern of contractile differentiation. Gestational MET treatment ablated these effects.

For effects between the MUN and CD groups, the MUN diet resulted in significantly greater colocalization values in the High-High category for normalized MUN Untreated-Sham arteries (Figure 4D). This indicated a significant increase in the most differentiated and contractile population. Relative to CD arteries, normalized Untreated-HI arteries from MUN pups exhibited colocalization values that were significantly less in the Low-Low category and significantly greater in both the Low-High and High-High categories, reflective of a rightward shift in pixel distribution and contractile differentiation. Arteries from the normalized MET-Sham group portrayed values of colocalization that were significantly less in the Low-Low category and significantly greater in the High-High category relative to CD arteries, indicative of a rightward shift and contractile differentiation. Colocalization values in normalized MET-HI results were significantly greater than CD values in the High-High category, which also implied greater contractile differentiation.

For comparisons between normalized MUN groups, mild HI resulted in colocalization values that were significantly less in the Low-Low category of Untreated-Sham arteries than values of Untreated-HI arteries, which implied a reduction in the least differentiated and least mature vSMC population. To summarize, in MUN arteries, mild HI promoted contractile differentiation through increased colocalization of αActin with SM-MHC, while preserving patterns of colocalization of αActin with NM-MHC through corticosteroid-dependent mechanisms. Additionally, relative to the Control Diet, the MUN diet generally promoted contractile differentiation and unmasked the ability of hypoxia to promote colocalization in arteries not transformed by gestational MET.

### 2.4. Passive Diameter and Compliance

**Figure 5:**
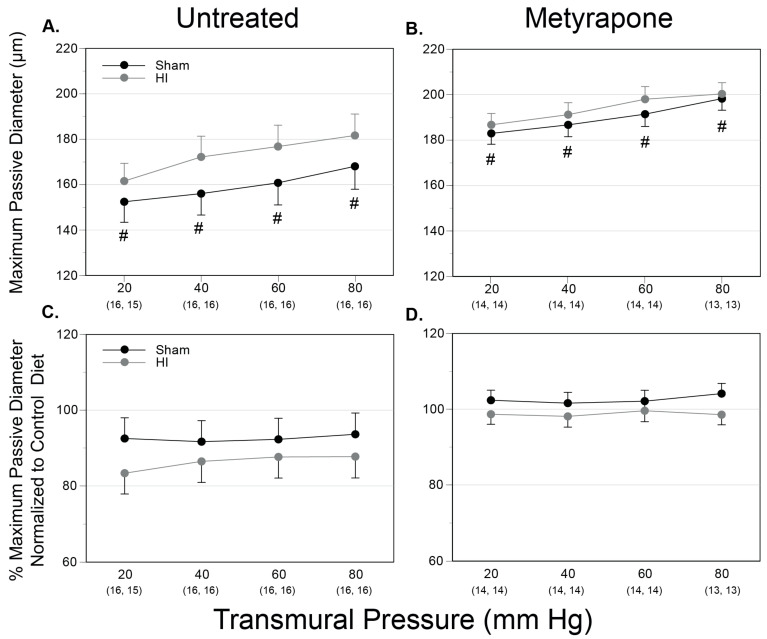
Effects of HI, gestational MET, and MUN on passive arterial diameter. (**A**,**B**) Incubation in 3 mM EGTA buffer eliminated all active tone in middle cerebral arteries from P11 pups and enabled the production of plots of passive vessel diameter against transmural pressure. (**C**,**D**) Each pressure step displays normalized values of MUN relative to CD to show MUN-induced effects. ANOVA with repeated measures combined with a post-hoc Fisher’s LSD determined the statistical significance as shown by: # = Untreated-Sham vs. MET-Sham. Parentheses at the bottom of each graph display *n* values for each group.

Across all MUN groups, the average passive outer middle cerebral artery diameter ranged from 152 ± 9 µm at 20 mm Hg to 200 ± 5 µm at 80 mm Hg (Figure 5A,B). For passive diameter measurements, these methods could detect a difference of 6.6% with a power of ≥0.8 for the total number of pups used (*n* = 60). For comparisons between MUN groups, passive diameters averaged to significantly greater values in MUN MET-Sham arteries compared to MUN Untreated-Sham arteries. Comparisons between the MUN and CD groups revealed no significant differences. Overall, these results demonstrate that gestational MET increased passive diameter and MUN did not.

### 2.5. Vessel Myography

**Figure 6:**
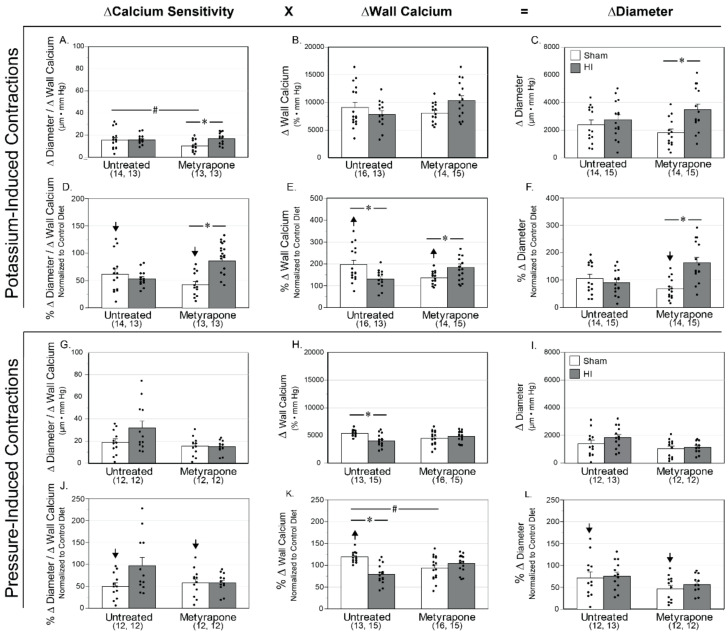
Effects of HI, gestational MET, and MUN on changes in diameter, wall calcium, and calcium sensitivity induced by K+ and pressure in MCA in P11/P12 pups. Middle cerebral artery measurements of contractile responses to changes in intraluminal pressure enabled assessment of responses in PSS with 120 mM K+ (**A–F**) and in PSS alone (**G–L**). Plots of these changes against intraluminal pressure on the abscissa allowed calculation of the areas beneath the curves for three different parameters on the ordinate: outside diameter (Δ Diameter), which is a product of wall calcium concentration (Δ wall calcium) and calcium sensitivity (Δ Diameter/Δ wall calcium). (**A–C**,**G–I**) Columns and error bars indicate SEM overlaid with a dot plot of individual values and show difference between MUN groups. (**D–F**,**J–L**) Columns and error bars indicate MUN values normalized to CD for each response and show MUN-induced effects. ANOVA with a post-hoc Fisher’s LSD determined the statistical significance as shown by: * for differences between Sham and HI groups and # for differences between MET-Sham and Untreated-Sham groups. Refer to Figure 4 for a description of the statistical symbols and *n* values shown above.

Measurements of potassium-induced active changes in middle cerebral artery diameter (∆D) enabled assessment of maximum reactivity from 20 to 80 mm Hg. Treatment with 120 mM potassium produced depolarization-induced contractility, which reflected maximum receptor-independent contractile capacity. Plots of these changes against intraluminal pressure allowed calculation of the areas beneath the ∆D–pressure curves (AUC), which in turn facilitated group-wise comparisons. Overall, the product of myofilament calcium sensitivity and intracellular calcium concentration determines contractile tone, [44], as indicated at the top of Figure 6. The methods used to estimate myofilament calcium sensitivity, calculated as the ratios of ΔD/Δ wall calcium could detect a difference of 28.2% with a power of ≥0.8 for the number of pups used (*n* = 53). The methods used to measure intracellular wall calcium could detect a difference of 10.0% with a power of ≥0.8 for areas beneath the ∆ wall calcium–pressure curves (AUC) for the number of pups used (*n* = 58). For diameter measurements, these methods could detect a difference of 25.2% with a power of ≥0.8 for the total number of pups used (*n* = 58). Whereas mild HI produced no change in any parameter between MUN Untreated-Sham and MUN Untreated-HI MUN groups, MET-HI arteries exhibited significantly greater changes in myofilament calcium sensitivity resulting in increased ∆D when compared to MUN MET-Sham arteries (Figure 6A–C). Additionally, gestational MET resulted in significantly lower myofilament calcium sensitivity in MUN MET-Sham pups relative to MUN Untreated-Sham pups (Figure 6A).

Relative to CD arteries, MUN produced values of myofilament calcium sensitivity that were significantly less for Untreated-Sham arteries and significantly greater for wall calcium with no significant changes in ∆D (Figure 6D–F). Relative to CD arteries, MET-Sham arteries displayed significantly lower myofilament calcium sensitivity along with significantly greater values of wall calcium, which produced values of potassium-induced ∆D that were significantly less than values for CD arteries. For effects between normalized MUN arteries, Untreated-HI arteries displayed values of wall calcium that were significantly less than in the Untreated-Sham arteries (Figure 6E).

The result of these individual changes produced patterns between normalized groups that describe the effect of the MUN diet on gestational MET transformation and neonatal HI responses. Normalized Untreated-HI arteries displayed values of wall calcium that were significantly less than values from normalized Untreated-Sham arteries. Values of myofilament calcium sensitivity and wall calcium in normalized MET-HI arteries were significantly greater than values in normalized MET-Sham arteries, which resulted in greater values of potassium-induced ∆D (Figure 6D,F). This same pattern emerged between normalized MUN MET-HI arteries and normalized MUN Untreated-HI arteries.

To complement measurements of maximum contractile reactivity, measurements of pressure-induced contractions in physiological saline solution enabled the study of myogenic reactivity, which reflected physiological activation of smooth muscle contraction (Figure 6G–L). For groupwise comparisons between MUN groups, mild HI produced values of ∆ wall calcium that were significantly less in arteries from the MUN Untreated-HI group than in MUN Untreated-Sham arteries (Figure 6H).

Relative to the Control Diet, the MUN diet produced values of myofilament calcium sensitivity that were significantly less and values of wall calcium that were significantly greater in Untreated-Sham arteries. In parallel, normalized values of pressured-induced ΔD were significantly less in Untreated-Sham arteries (Figure 6J–L). Normalized MUN MET-Sham arteries yielded values that were significantly less for myofilament calcium sensitivity and pressure-induced ΔD.

Groupwise comparisons of these individual changes showed that in normalized MUN arteries mild HI produced values of wall calcium mobilization that were significantly less in Untreated-HI arteries than in Untreated-Sham arteries (Figure 6K). Additionally, normalized wall calcium mobilization values were significantly less following gestational MET treatment than in the Untreated-Sham arteries. In summary, these results demonstrate a fundamental ability of gestational MET to attenuate myogenic reactivity. Additionally, relative to values from the CD group, the MUN values were significantly less for myofilament calcium sensitivity. Owing to the fact that changes in artery diameter are determined largely by the product of myofilament calcium sensitivity and calcium mobilization (see top of Figure 6), the observed changes in myofilament calcium sensitivity help explain the smaller corresponding changes in ∆diameter.

### 2.6. MRI

**Figure 7:**
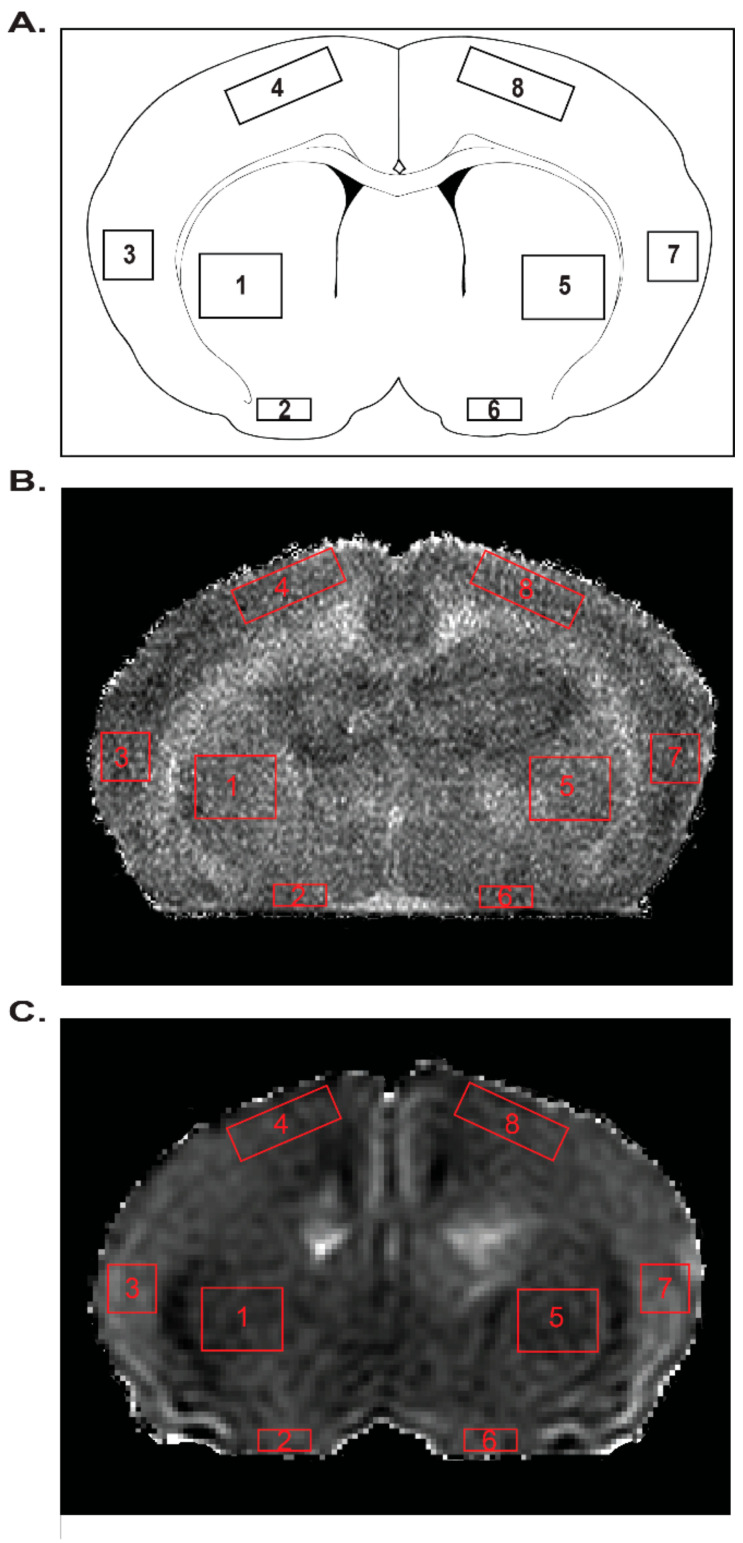
(**A**) displays the location of each of the four regions of interest in each hemisphere, as marked by numbered red boxes on a coronal section from an anatomical map at Bregma level 0.20 mm. Regions of interest 1–4 represent the left striatum, ventral cortex, lateral cortex, and dorsal cortex, respectively, whereas regions of interest 5–8 represent the right striatum, ventral cortex, lateral cortex, and dorsal cortex, respectively. (**B**) shows a T2 map and (**C**) an ADC map from the brain of a P11 MUN Untreated-Sham rat.

**Figure 8:**
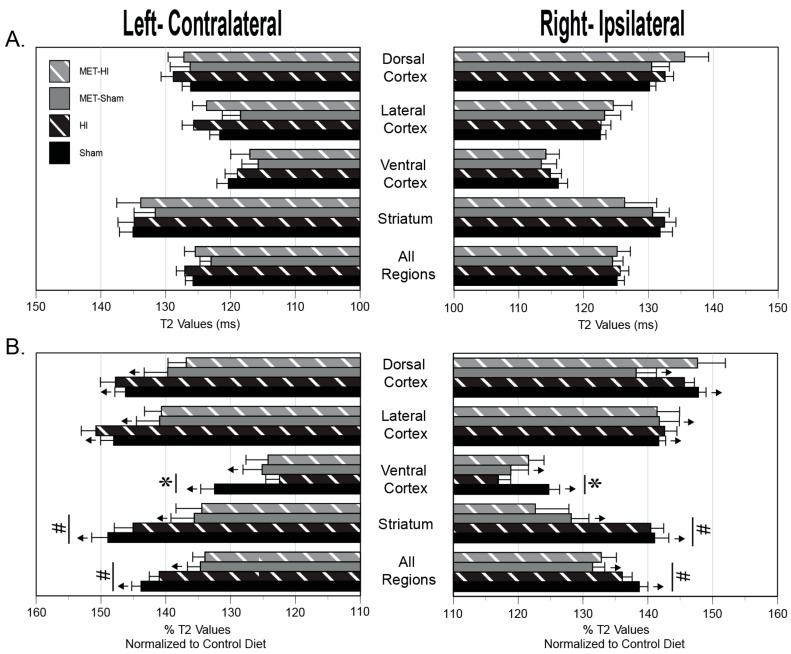
Effects of HI, gestational MET, and MUN on T2 values in P11/P12 pups. Regions of interest from four regions in each hemisphere are depicted above. The “All Regions” results represent hemispheric averages of the four regions combined. (**A**) Columns and error bars indicate SEM and show difference between MUN groups. (**B**) Columns and error bars indicate MUN values normalized to CD for each response and show MUN-induced effects. ANOVA with a post-hoc Fisher’s LSD determined the statistical significance as shown by: * for differences between Sham and HI groups and # for differences between MET-Sham and Untreated-Sham groups. Refer to Figure 4 for a description of the statistical symbols shown above. *n* = 12 in all groups except MET-Sham where *n* = 11.

**Figure 9:**
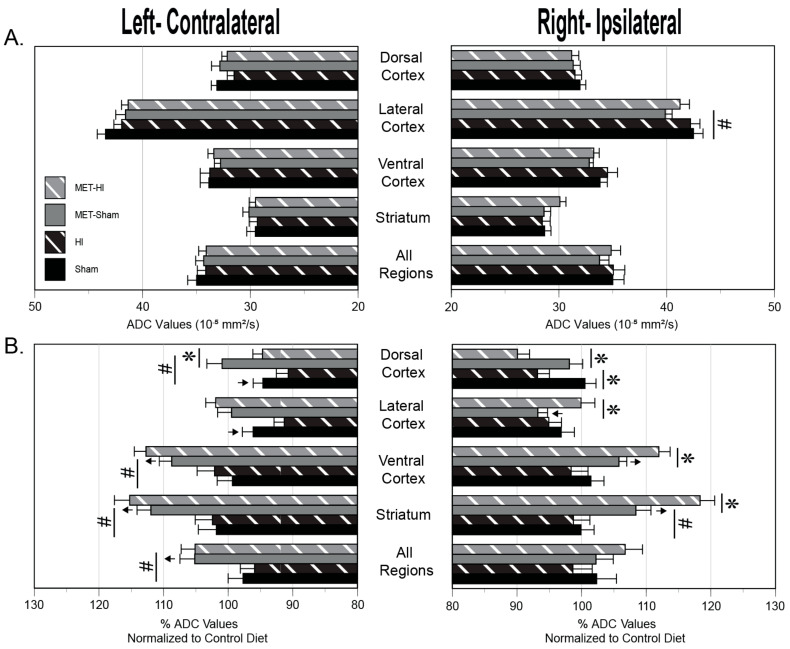
Effects of HI, gestational MET, and MUN on ADC values in P11/P12 pups. Regional analysis of apparent diffusion coefficients (ADC) used the same regions and layout as Figure 8. (**A**) Columns and error bars indicate SEM and show difference between MUN groups. (**B**) Columns and error bars indicate MUN values normalized to CD for each response and show MUN-induced effects. ANOVA with a post-hoc Fisher’s LSD determined the statistical significance as shown by: * for differences between Sham and HI groups and # for differences between MET-Sham and Untreated-Sham groups.

Analysis of T2 and ADC values portray the effects of mild neonatal HI, gestational MET transformation and MUN programming on neonatal cerebral tissue integrity four regions of interest in each hemisphere (Figure 7, Figure 8 and Figure 9). The methods used to measure these results could detect a difference of 3.3% (T2) and 6.2% (ADC) with a power of ≥0.8 for the number of pups used (*n* = 47). For comparisons between MUN groups, T2 values, which generally identify cerebral edema, did not significantly differ between any group (Figure 8A), confirming the very mild nature of the HI.

In contrast, relative to results from the Control Diet group, MUN brains exhibited significantly greater T2 levels for every group in each region surveyed (Figure 8B). Regarding effects between the normalized MUN groups, T2 values in the Untreated-HI group were significantly less than in the Untreated-Sham group in both the left and right ventral cortex (Figure 8B). Gestational MET transformation resulted in T2 values that were significantly lower in the normalized MUN MET-Sham group than in the normalized MUN Untreated-Sham group in both the left and right All Regions averages and the left and right striatum.

For comparisons between MUN groups, statistical analysis of ADC values, which typically indicate changes in water mobility and cell swelling, revealed a significant gestational MET-induced depression in MUN MET-Sham ADC values relative to MUN Untreated-Sham values in the right lateral cortex (Figure 9A). Regarding the effect of the MUN diet relative to Control Diet, MUN produced ADC values that were significantly less than CD values in both the left lateral and dorsal cortex regions (Figure 9B). Additionally, normalized MET-Sham ADC values were significantly greater than CD values in the left All Regions average, the left and right striatum, and the left and right ventral cortex. Normalized Met-Sham ADC values also were significantly less than CD values in the right lateral cortex.

For comparisons between the normalized MUN groups, mild hypoxia produced ADC values that were less in the normalized Untreated-HI group than in normalized Untreated-Sham of the right dorsal cortex (Figure 9B). Mild HI also resulted in ADC values that were significantly less in the normalized MET-HI group than in the normalized MET-Sham group in the right and left dorsal cortex. Additionally, ADC values were significantly greater in the normalized MUN MET-HI group than in the normalized MET-Sham group in the right striatum, right ventral cortex, and right lateral cortex. Regarding changes in the normalized MUN MET-Sham group relative to the normalized MUN Untreated-Sham group, ADC values were significantly greater in the left All Regions average, left and right striatum, left ventral cortex, and left dorsal cortex.

In aggregate, between the MUN groups, mild HI and gestational MET programming had no effect on edema formation or cellular swelling, with the exception of increased cellular swelling in the right lateral cortex in response to gestational MET programming. However, compared to Control Diet, the MUN diet produced global edema with a heterogenous pattern of hypoxic and gestational MET-programmed effects. Further, relative to the Control Diet, the MUN diet resulted in a region-specific pattern of altered levels of cellular swelling, as well as a diverse pattern of effects brought on by mild HI and gestational MET.

### 2.7. Neurobehavior

**Figure 10:**
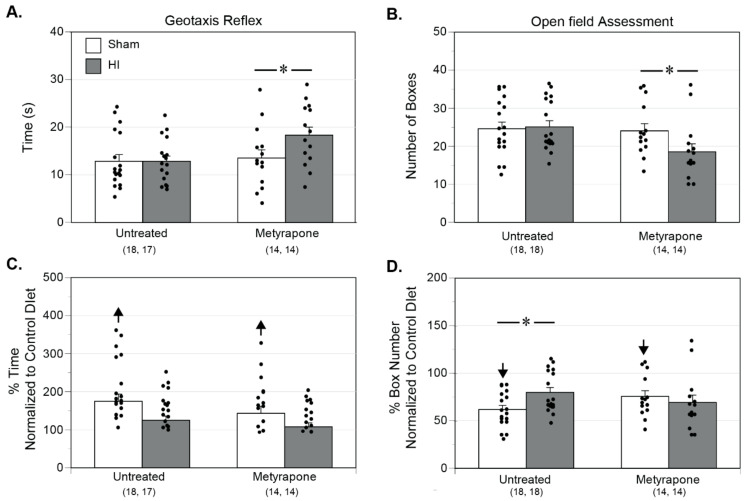
Effects of HI, gestational MET, and MUN on neurobehavioral measures in P11/P12 pups. Neurobehavioral assessments conducted in neonatal MUN rats examined the geotaxis response (**A**,**C**) and open-field exploration (**B**,**D**). (**A**,**B**) Columns and error bars indicate SEM overlaid with a dot plot of individual values and show difference between MUN groups. (**C**,**D**) Columns and error bars indicate MUN values normalized to CD for each response and show MUN-induced effects. ANOVA with a post-hoc Fisher’s LSD determined the statistical significance as shown by: * for differences between Sham and HI groups. Refer to Figure 4 for a description of the statistical symbols and *n* values shown above.

Across the neurobehavioral assessments used in P11/P12 pups, the statistical sensitivity of the geotaxis reflex test could detect differences of 14.8% with a power of ≥0.8 for the number of pups used (*n* = 63). For comparisons of raw data between MUN groups, gestational MET transformation unmasked an HI-induced increase in the time required to perform the geotaxis reflex in MET-treated pups (Figure 10A). Relative to the Control Diet, the MUN results revealed that a significantly greater amount of time was needed to turn 180° in pups from the normalized Untreated-Sham and MET-Sham groups (up arrows, Figure 10C).

The open-field assessment could detect differences of 11.0% with a power of ≥0.8 for the number of pups used (*n* = 64). Between MUN groups, gestational MET unmasked a significant reduction in exploration in MUN MET-HI pups relative to MUN MET-Sham pups (Figure 10B). Concerning comparisons between the MUN diet and the Control Diet, MUN significantly attenuated exploration in all normalized groups (down arrows, Figure 10D). Interestingly, the number of boxes explored was significantly greater in pups from the normalized Untreated-HI group than for pups in the normalized Untreated-Sham group. To summarize, these results demonstrate a fundamental role of corticosteroids in maintaining the geotaxis reflex and exploratory behavior following HI injury. Additionally, MUN generally worsened behavioral outcome and, with regards to exploration, introduced a corticosteroid-dependent effect of HI.

## 3. Discussion

The present study explored the hypothesis that an adverse intrauterine environment caused by maternal undernutrition acted through corticosteroid-dependent and -independent mechanisms to program lasting functional changes in the neonatal cerebrovasculature. To address the possibility of sexual dimorphic differences among the endpoints measured [45], all data underwent statistical analyses designed to detect male-female differences. These analyses revealed no sex-dependent differences, and thus all reported statistical comparisons relied on pooled data from male and female rat pups, as previously reported [35]. Overall, this study examined two fundamental questions regarding the effects of a restricted maternal diet during late gestation, on neonatal cerebrovascular consequences evident at cellular, tissue, whole brain, and organismal levels. The first question evaluated the lasting effects of maternal undernutrition on baseline cerebral and cerebrovascular characteristics, and the involvement of gestational corticosteroids in these effects. The second question assessed the influences of MUN on neonatal cerebrovascular responses to mild hypoxia-ischemic injury, and the contributions of gestational corticosteroids to alteration of these responses. In aggregate, the results demonstrated that chronic fetal stress induced by maternal undernutrition modulated neonatal cerebral and cerebrovascular responses through both corticosteroid-dependent and -independent mechanisms.

A broad variety of studies in many different preparations have clearly demonstrated that MUN can permanently alter function of the offspring HPA axis, although experimental conditions, animal age, type and severity of stressor, and length and severity of undernutrition all determine the impact of HPA axis modulation [26,46]. To assess the importance of this effect in our P9–P12 rat pups, the present study assessed the lasting impact of MUN on baseline and stress response levels of total plasma corticosterone. At 24 h after Sham treatment (Figure 1, Endpoint 3), MUN produced significantly greater values of total plasma corticosterone concentration than in pups from dams fed our Control Diet (Figure 2F), suggesting increased synthesis and/or decreased clearance of corticosterone. Factors driving increased synthesis could include increased sensitivity to releasing hormones [47], Sham treatment [48], or alternatively, decreased negative feedback at the pituitary and hypothalamus [26]. Whereas published evidence suggests that the HPA axis in neonatal rats is typically hyporesponsive to stress [49], it remains possible that MUN could truncate or ablate this hyporesponsive interval. In addition, decreased clearance of corticosterone has previously been reported in response to fasting [50], which could be attributable to decreased corticosterone catabolism or increased expression of corticosterone binding globulin [51]. Better understanding of the mechanisms whereby MUN alters the HPA axis will require further experiments to determine how MUN influences circulating ACTH levels, responses to exogenous ACTH, clearance of exogenously administered corticosterone and its feedback effects on ACTH levels, as well as glucocorticoid receptor levels in many different tissues.

To help elucidate how changes in gestational glucocorticoid levels might contribute to the effects of MUN, the present study employed MET, a potent inhibitor of corticosteroid synthesis [29] as we have previously described [27,36]. Gestational MET resulted in significantly lower values of basal corticosterone in naïve P9/P10 pups (Figure 2D), and in P10/P11 pups 2 h after Sham treatment (Figure 2E), but not in P11/P12 pups 24 h after Sham treatment (Figure 2F), demonstrating that gestational MET produces a transient attenuation of circulating corticosterone levels in postnatal rats. The corticosterone data imply that sustained effects of MET observed at 24 h post-Sham treatment for other endpoints cannot be attributed to any significant effect of MET on circulating corticosterone (Figure 2C), and instead arise from other factors such as differences in corticosteroid receptor levels. MET can also influence the levels of corticosteroids other than corticosterone, the most important of which is probably aldosterone [28]. In turn, attenuation of aldosterone synthesis by MET could affect smooth muscle differentiation through both direct effects on mineralocorticoid receptors [52], and indirect effects on levels of angiotensin II, a potent modulator of smooth muscle phenotype [53]. Whereas our corticosterone data do not illuminate how MUN and MET independently influenced the HPA in our rat pup model, the data do demonstrate that gestational MUN, but not MET, significantly altered adrenal production of corticosterone in P11/P12 pups (Figure 2F), which in turn may have influenced the cerebral and cerebrovascular endpoints measured.

Given that both MUN and corticosteroids influence the differentiation and maturation of vascular smooth muscle [54,55] the present studies examined the effects of MUN on the phenotype of neonatal cerebrovascular smooth muscle. Typically, immature vascular smooth muscle expresses αActin and NM-MHC to produce a low capacity contractile phenotype [32,34]. With maturation and contractile differentiation, αActin continues to be abundantly expressed but with an increasing proportion of SM-MHC in place of NM-MHC [34]. MUN during late gestation produced a programming effect evident in P11/P12 neonates that increased cerebrovascular smooth muscle contractile differentiation, as revealed by increased NM-MHC and SM-MHC colocalization with αActin (Figure 4B,D). This finding, together with previous reports that expression of non-muscle myosin heavy chain decreases with vascular maturation [32,34], emphasizes that contractile protein abundance and colocalization are not always tightly coupled [56,57]. Simultaneous administration of MET during late gestation MUN significantly enhanced αActin colocalization with both SM-MHC and NM-MHC (Figure 4B,D), revealing a lasting, but modest effect of corticosteroids on basal levels of smooth muscle differentiation in neonatal cerebral arteries. Altogether, the colocalization results imply that MUN potently enhanced contractile differentiation that was limited by corticosteroid-dependent mechanisms (Figure 4B), and mildly enhanced contractile differentiation through corticosteroid-dependent and -independent mechanisms (Figure 4D).

Owing to the close relations among smooth muscle phenotype, vascular structure, and function [33,58], the present studies examined the influences of MUN and MET on maximum passive diameter in neonatal middle cerebral arteries. Whereas MUN did not affect passive diameter (Figure 5C,D), MET treatment significantly increased diameters (Figure 5A,B), implying that corticosteroids promoted smaller artery diameters during development, perhaps through promotion of contractile differentiation (Figure 4B) or inhibition of extracellular matrix content of collagen and elastin [59]. Regarding contractile behavior in neonatal arteries, MUN also enhanced depolarization-induced calcium mobilization, leading to increases in cytosolic calcium concentration, presumably via enhanced entry through sarcolemmal voltage-dependent calcium channels [58]. MUN also depressed myofilament calcium sensitivity that offset changes in calcium mobilization, resulting in no significant change in artery diameter (Figure 6D–F). Similarly, MUN enhanced stretch-induced (myogenic) calcium mobilization and attenuated myofilament calcium sensitivity, which in combination, slightly but significantly, decreased artery diameter (Figure 6J–L). Regarding the effects of corticosteroids on these patterns, treatment with MET during MUN did not affect depolarization-induced contractions (Figure 6D–F), but unmasked values of calcium mobilization for stretch-induced contractions that were less than CD values without affecting changes in artery diameter (Figure 6J–L). Together, these results imply that MUN attenuated myofilament calcium sensitivity through corticosteroid-independent mechanisms, enhanced calcium mobilization through corticosteroid-dependent mechanisms evident during stretch-induced, but not during depolarization-induced, contractions, but exerted only modest effects on changes in diameter depending on the method of contraction. In turn, the greater effects of MUN and MET on calcium mobilization and diameter during submaximal stretch-induced contractions, compared to maximal depolarization-induced contractions, predict competitive inhibitory effects of MUN and MET on calcium-dependent contractility. Further experiments are needed to identify which mechanisms, including possible modulation of the ion channels and pumps that govern cytosolic calcium concentration [58] and the enzymes such as Rho-Kinase, MLCK and MLCP that determine myofilament calcium sensitivity [60], contribute most to the vascular effects of MUN.

At the level of whole brain MRI, MUN caused global increases in T2 values, indicating modest increases in edema (Figure 8B). This increased edema, in turn, could have arisen from increased blood–brain barrier permeability [61,62] or delayed cerebrovascular maturation caused by lasting postnatal effects of MUN [63]. In turn, MUN could have potentially delayed blood–brain barrier development by attenuating expression of tight junction proteins such as claudins and occludins, which can be depressed in some [64] but not all [65,66] situations by corticosteroids. In addition, corticosteroids can also reduce brain weight and myelination, which may also elevate T2 signals and presumably brain water content [63,67]. Consistent with a possible corticosteroid-dependent ability of MUN to increase blood–brain barrier permeability, treatment with MET during MUN attenuated postnatal T2 values throughout the brain, and particularly in the striatum (Figure 8B). To differentiate between vasogenic or cytotoxic edema, the MRI protocols completed also included calculations of ADC values to assess changes in cell volume and swelling. Relative to values from CD pups, MUN produced ADC values that were modestly but significantly less than CD values in the left dorsal and lateral cortex, suggesting increased swelling in these areas [68] (Figure 9B). In parallel with the T2 results, treatment with MET during MUN increased ADC values throughout the left side of the brain and also in the right striatum, indicating cell shrinkage due possibly to cell autophagy, apoptosis, or death [69]. Altogether, the MRI results demonstrated that MUN yielded global T2 values that were greater than CD values without widespread changes in cell volume, suggesting vasogenic edema possibly secondary to increased blood brain barrier permeability. Treatment with MET during MUN modestly attenuated edema formation, indicating that corticosteroids contributed, in part, to the heterogeneous effects of MUN on neonatal cerebral edema.

In light of the many observed effects of MUN on the structural and functional maturation of the cerebrovasculature, our experimental approach also examined neurobehavior as an index of integrated cerebral function. MUN pups required a greater amount of time to complete the geotaxis reflex in a corticosteroid-independent manner, indicating impaired motor coordination (Figure 10C), due possibly to compromised vestibular input [70]. MUN also worsened performance in a corticosteroid-independent manner during an open-field assessment (Figure 10D), indicating reduced exploration and locomotion, possibly due to anxiety [71] or compromised neuronal function [72]. Consistent with the effects of MUN on cerebral arteries and edema, our model of MUN impaired neurobehavioral performance consistent with other studies [73,74], and through mechanisms independent of gestational changes in corticosteroids.

In addition to its focus on the corticosteroid-dependent and -independent effects of MUN on baseline cerebrovascular characteristics and neurobehavior, our experimental design also examined how MUN and corticosteroids influenced cerebral responses to hypoxic ischemia, a common cardiovascular stressor in neonates. In particular, our model evaluated mild hypoxic-ischemic injury [35], which is more common clinically than the widespread hemispheric injury produced by other neonatal models of more severe hypoxic-ischemic injury [41]. Consistent with other studies of neonatal stress [75,76], our model of mild hypoxic ischemia increased corticosterone levels 2 h after HI injury (Figure 2E,F), but this effect dissipated by 24 h. MET treatment ablated the increase in corticosterone values at 2 h post-hypoxia, but had no effect at 24 h, suggesting that MUN programmed a robust but transient, MET-sensitive increase in corticosterone following mild HI injury. The absence of significant effects of HI on corticosterone at 24 h in either MET-treated or untreated pups, further implies that any effects of HI observed at 24 h cannot be attributed to changes in circulating corticosterone levels (Figure 2C).

Consistent with previous studies [35,37], neonatal hypoxic ischemia significantly shifted the phenotype of cerebrovascular smooth muscle (Figure 4B,D). In pups programmed by MUN, mild HI insult promoted contractile differentiation, as indicated by a rightward shift in pixel distributions for αActin colocalization that was most prominent with NM-MHC, but was also significant with SM-MHC (Figure 4B,D). In pups programmed by both MUN and MET, the effects of HI depended markedly on the MHC isoform (Figure 4B,D). For colocalization of αActin with NM-MHC, treatment with MET indicated that corticosteroids inhibit differentiation in poorly differentiated cells (Low MHC/Low αActin) but promoted contractile differentiation in highly differentiated cells (High MHC/High αActin). For colocalization of αActin with SM-MHC, in contrast, treatment with MET revealed that corticosteroids mildly promoted contractile differentiation following HI; in MET-treated MUN pups, HI did not affect colocalization of αActin with SM-MHC in any category. For both MHC isoforms, corticosteroid reduction during gestation programmed lasting sensitivity of cerebrovascular smooth muscle phenotype to the effects of HI, perhaps through loss of local factors that sustain differentiation and gain of factors that promote proliferative and migratory pathways [58]. The aggregate results demonstrate that MUN programming enhanced the ability of HI to promote contractile differentiation, and that gestational corticosteroids contributed to this general effect more for NM-MHC than for SM-MHC.

To assess the functional consequences of altered smooth muscle phenotype, the experimental protocols included measurements of arterial passive diameter and contractility in arteries from MUN pups exposed to mild HI. In contrast to its effects on smooth muscle phenotype (Figure 4), HI did not alter the passive diameters of MUN pups from MUN dams treated with or without MET (Figure 5C,D). Regarding contractility, however, MUN programming enhanced HI-induced depression of calcium mobilization without significant changes in artery diameter in both depolarization- and pressure-induced contractions (Figure 6E,F,K,L). This pattern of effects could reflect lasting consequences of MUN programming on cytosolic calcium regulation via either attenuation of intracellular release or extracellular influx, or conversely, via increased intracellular sequestration or extracellular extrusion of calcium [77]. Although HI-induced changes in calcium mobilization did not affect the diameter of arteries from MUN-programmed pups, these changes still may have altered calcium-dependent gene expression, and thereby altered smooth muscle phenotype, as reported in other studies [78]; our current findings (Figure 4) support such an effect. MET treatment reversed or ablated HI-induced changes in calcium mobilization, supporting the idea that these changes in HI handling involved corticosteroid-dependent mechanisms (Figure 6E,K). MET treatment unmasked HI enhancement of depolarization-induced increases in artery diameter secondary to increases in myofilament calcium sensitivity and wall calcium (Figure 6D–F), suggesting that gestational corticosteroids inhibited HI-induced increases in calcium mobilization and myofilament sensitivity. On the whole, MUN programming enhanced HI-induced depression of calcium mobilization in pup cerebral arteries regardless of method of contraction, and these effects required gestational corticosteroids.

At the level of the whole brain, MUN programming caused HI-induced reductions in T2 values that were significantly less than CD values only in the right and left ventral cortex, consistent with a region-specific reduction in edema (Figure 8B). These decreases in T2 values could arise from HI-induced improvement of blood–brain barrier or increased sodium clearance through enhanced ion pump function, as previously suggested [79]. Alternatively, reduced vasogenic edema could result from decreased blood flow in the ventral cortex [80], which lies closest to the Circle of Willis among the regions examined, and could reflect preferential sensitivity to carotid ligation in our model. MUN programming also enhanced HI-induced decreases in ADC values that were significantly less than CD values, implying increased cellular swelling, in the right dorsal cortex (Figure 9B), consistent with regional energy failure [81] secondary to compromises in either local perfusion or the capacity for ATP synthesis. Gestational MET eliminated the effects of HI on T2 values in the ventral cortices of MUN pups (Figure 8B), indicating that MUN produced a regionally heterogeneous pattern of HI-induced attenuation of corticosteroid-dependent edema formation. In addition, gestational MET during MUN produced a very heterogeneous pattern of effects in which corticosteroids appeared to inhibit HI-induced cell swelling in the left but not right dorsal cortex, opposed HI-induced cell shrinkage in the right lateral cortex, ventral cortex, and striatum, but did not affect HI-induced changes in edema and cell volume in all other regions (Figure 9B). These patterns strongly imply that both MUN and MET treatment caused significant region-specific cerebral differences in expression of genomic and/or non-genomic corticosteroid receptors [25,26].

To assess the integrated consequences of MUN and HI at the cellular, tissue, and whole brain levels of organization, separate experiments quantified the effects of HI on geotaxis and open-field behavior. In MUN-programmed pups, HI did not alter geotaxis performance but improved exploratory behavior (Figure 10D), suggesting HI-induced hyperactivity as reported in other studies [72]. In turn, the enhanced locomotion may have arisen from MUN-programmed HI-induced reductions in edema in the ventral cortices (Figure 8B), consistent with findings in other studies [82,83]. The effects of MET treatment during MUN (Figure 10D) further suggest that gestational corticosteroids in some way enabled the effects of HI on open-field behavior, perhaps by attenuation of HI-induced decreases in ventral cortex edema (Figure 8B). Overall, the neurobehavioral results show that, following mild HI, MUN programming altered pathways involved in exploration and motor control, but not those involved in the geotaxis reflex, through corticosteroid-dependent mechanisms.

## 4. Materials and Methods

### 4.1. General Preparation

The Loma Linda University Institutional Animal Care and Use Committee approved all experimental procedures used in these studies (IACUC Protocol# 8190025, approved on 9 October 2019). The Animal Care Facility housed pregnant Sprague–Dawley rats (Charles River Laboratories, Hollister) at constant temperature and humidity with a 12:12 h:light–dark cycle. From day 10 of gestation until term (21 days), maternal undernutrition (MUN) and MUN-metyrapone (MUN-MET) group rats consumed a diet of standard laboratory chow (Lab Diet 5001, Brentwood, MO: protein 23%, fat 4.5%, metabolizable energy 3030 kcal/kg) restricted to 50% of the intake measured by weight in paired ad libitum fed Control Diet (CD) rats. On gestational day 11, approximately half of the rats’ drinking water contained MET (cat# 14,994 Cayman Chemical Company, Ann Arbor, MI, USA), freshly dissolved each day at a final concentration of 0.5 mg/mL (Figure 1). Previous studies have demonstrated this concentration of MET to block maternal corticosterone synthesis and significantly reduce plasma corticosterone levels in both fetal and neonatal blood [27]. Daily water consumption did not differ between Control (standard filtered water) and MET-treated dams. All rats delivered spontaneously, and pups remained with their dams until the day of surgery. The overall study design included four MUN groups of Sprague–Dawley rats: (1) Sham-operated animals (MUN Untreated-Sham), (2) animals that underwent unilateral carotid ligation surgery followed by hypoxia 24 h later (MUN Untreated-HI), (3) Sham animals that received MET during gestation (MUN MET-Sham), and (4) HI animals that received MET during gestation (MUN MET-HI). Additionally, comparisons between these novel MUN groups and a previously published cohort of Control Diet counterpart groups enabled the study of the effects of MUN relative to CD [35].

### 4.2. Blood Collection and Corticosterone Analysis

A time course study of corticosterone levels relative to the timing of the hypoxic insult quantified changes in corticosterone levels in each of the experimental groups (Figure 1). The collection of whole trunk blood into Lithium Heparin blood collection tubes (Greiner Bio-One, Kremsmunster, Austria) occurred at the time of sacrifice, according to three time course endpoints: immediately before surgery (pretreatment baseline; GC Endpoint 1); 2 h after HI injury (early ischemic endpoint; GC Endpoint 2); and 24 h after HI injury (late ischemic endpoint; GC Endpoint 3). To reduce variability due to circadian oscillations in corticosterone levels, blood collection always occurred during a 4 h block in the morning. Plasma samples then underwent centrifugation at 2000 RPM at 4 °C for 15 min to yield aliquots frozen at −20 °C.

In preparation for ELISA assays, the sample extraction procedure included treatment with perchloric acid followed by potassium hydroxide, which released any corticosterone bound by protein in thawed plasma samples. Twice-repeated additions of ethyl acetate followed by centrifugation extracted corticosterone in the samples into the supernatant. A Savant SpeedVac Concentrator (ThermoFisher Scientific, Waltham, MA, USA), then evaporated the supernatants. This step of the extraction concluded with capping and freezing the dried samples at −80 °C until the time of assay.

Corticosterone quantification employed an ELISA kit (cat#: ADI-900-097, ENZO Life Sciences, Farmingdale, NY, USA) and a Gen5 1.11 BioTek microplate reader to read the samples (run in duplicate) at 405 nm. Known standards, provided, and run with each kit, enabled construction of standard curves that determined the quantity of corticosterone in each sample in picograms per milliliter. The kits detected a minimum concentration of 27 pg/mL and provided intra-assay variations no greater than 4%.

### 4.3. Carotid Ligation and Hypoxic Exposure: A Model of Mild Hypoxic Ischemia

On postnatal days 9 and 10 (P9, P10), which correspond to the brain development of a full-term human infant (39), pup littermates received surgical treatment (Figure 1). Following the induction of anesthesia with 3% isoflurane inhalation, the pups underwent right carotid ligation using 5-0 surgical silk (Ischemia surgery) or dissection without ligation (Shams). All surgeries typically lasted less than 15 min, which minimized the potential neuroprotective effects of isoflurane exposure (5). Postsurgical pups recovered with their dam for 24 h, after which pups designated for hypoxia experienced exposure to a commercially prepared mixture containing 8% oxygen with balance nitrogen for 90 min in a sealed, humidified chamber maintained at 37 °C. An oxygen monitor continuously displayed oxygen levels in the chamber to assure consistency of the hypoxic exposure. After hypoxic exposure, the pups recovered 15 min under normoxic conditions (room air) in the chamber, and then continued recovery with their dam. Sham pups, which had no carotid ligation, underwent exposure to normoxia (room air) for 90 min at 37 °C.

### 4.4. Confocal Microscopy

The confocal microscopy protocol used MCAs from separate cohorts of P11/P12 pups in the four treatment groups. Overnight fixation of these MCA segments in 4% PFA preceded the processing and embedding of arteries in paraffin and subsequent microtomy into 5 µm coronal sections. All IHC staining runs included a slide cut from the same standard block of a naïve cerebral artery. Double-staining the sections with antibodies against smooth muscle αactin (Sigma-Aldrich, St. Louis, MO, USA, A5228 @ 1:300 in PBS containing 2% NGS, 1% BSA at 4 °C for 18 h) and either smooth muscle myosin heavy chain (Abcam, Cambridge, UK, Ab53219 @ 1:300 in PBS containing 2% NGS, 1% BSA at 4 °C for 18 h) or non-muscle myosin heavy chain (BioLegend, San Diego, CA, USA, Poly19099 @ 1:300 in PBS containing 2% NGS, 1% BSA at 4 °C for 18 h) allowed for identification of smooth muscle phenotype. The visualization procedure used secondary antibodies (Dylight-488 and Dylight-633 @ 1:300) in PBS containing 2% NGS, 1% BSA incubated at 22 °C for 2 h. An Olympus FV1000 confocal microscope (Toyoko, Japan) produced coronal images using a lens with a numerical aperture of 1.4 to yield lateral resolutions between 146 and 185 nm, and axial resolutions between 545 and 693 nm, depending on the wavelengths of illumination. Z-stack images were captured at a thickness of 0.45 μm per slice, for a total of 10 slices per artery. Imaging always began with the standard section for the purpose of setting the key imaging parameters, including scan time, gain, illumination intensity, and photomultiplier voltage, to provide between 1% and 5% saturation, as confirmed off-line through Histogram analysis using FIJI software (ImageJ v2.1.0/1.53c). This allowed for the capture of all images under identical conditions. Analyses of the confocal images of the artery sections employed CoLocalizer Pro (Version 2.6.1, CoLocalization Research Software, Kochi, Japan) to calculate the extent of colocalization between the markers in each pair. For each artery, three slices from each stack (Slices 2, 5, and 7) underwent analysis. Each pixel (above a set threshold) in each double-stained image fell into one of three categories: Low Marker#1 and Low Marker #2, Low Marker #1 and High Marker #2, and High Marker #1 and High Marker #2 (Marker 1 represented one of the two myosin heavy chain isoforms and Marker 2 represented smooth muscle αactin). The analysis involved calculation of the number of colocalized pixels in each category as a percentage of the total number of colocalized pixels for both markers across all 3 categories (defined as 100%). Previous publications include detailed descriptions of all these methods [36,84] and validation by comparison to other established methods for quantitation of protein colocalization via confocal microscopy [85].

Validation of the titers for each of the antibodies used to image cerebrovascular contractile proteins relied on the results of immunoblots of PAGE gels performed with at least 5 different titers to probe standards prepared from homogenates of adult cerebral arteries. By definition, optimum primary titers identified only one major band. Optimum secondary antibody titers, by definition, produced a blank image after IHC staining in the presence of secondary but not primary antibodies. These approaches have been standard practice for many years [86,87].

### 4.5. Vessel Myography

To determine the contractile characteristics of MCAs, experiments with adjacent MCA artery segments included serial recordings of arterial diameter, first in physiological saline solution (PSS) and then in 120 mM K+ solution to determine maximum contractile capacity. Fura-2-AM (ThermoFisher Scientific, Waltham, MA USA) loaded at 1 µM, enabled simultaneous measurement of smooth muscle calcium concentration. The protocol included measurements in PSS and then 120 mM K+ at 20, 40, 60 and 80 mm Hg. After the final pressure step, addition of a zero calcium, 3 mM EGTA solution quenched cytosolic calcium to enable measurement of passive diameters at each pressure level used for contractility measurements. Previous publications detail all methods employed in these measurements [36,84].

### 4.6. Perfusion Fixation

Following behavioral testing (described subsequently), transcardial perfusion fixation enabled subsequent measurement of HI injury via MRI and histology. After the induction of anesthesia with 3% isoflurane inhalation, the perfusion procedure began with exposure of the heart followed by the passage of a 25-gauge butterfly needle through the left ventricle and into the aorta. A peristaltic pump (Model EP-1 Econo Pump; Bio Rad, Hercules, CA, USA) connected to the needle infused phosphate-buffered saline into the vasculature for the removal of blood through an incision in the right atrium. After this, perfusion with a solution containing 4% paraformaldehyde at 8 mL/min fixed the brain. The entire procedure, not including induction time, lasted approximately 15 min. Following perfusion fixation, post-fixation of the extracted brains occurred overnight in 4% PFA in PBS at 4 °C.

### 4.7. Postmortem MRI

An 11.7 T Bruker Avance instrument (Bruker Biospin, Billerica, MA, USA) generated images from ex vivo whole perfusion-fixed brains. As indicated by a growing number of studies, ex vivo MRI is highly useful for revealing changes in brain structure and water mobility [88]. Two imaging sequences (T2-weighted (T2WI) and diffusion weighted imaging (DWI)) assessed HI injury for changes in water content and water mobility, respectively. Each sequence collected 20 coronal slices with a slice thickness of 0.75 mm. The T2 sequence used the following parameters: TR/TE = 3000/10 ms, matrix = 192 × 192 zero-filled to 256 × 256 at reconstruction, field of view (FOV) = 1.5 cm, and a total of 2 averages. The DWI sequence employed these parameters: TR/TE = 5000/31.2 ms, b value = 200 s/mm^2^, matrix = 64 × 64 zero-filled to 128 × 128 at reconstruction, FOV = 1.5 cm, and a total of 2 averages. JIM, a medical imaging analysis software (Xinapse Systems Ltd.; West Bergholt, Essex, United Kingdom) generated T2 maps and processed the DWIs to produce ADC maps. Manually drawn regions of interest using FIJI sampled quantitative values in right (ipsilateral), left (contralateral to the side of carotid ligation), cortical, and striatal hemispheres for each slice of all ADC and T2 maps (Figure 7). For comparisons across groups, posterior/anterior analyses utilized the regional averages from the 13th or 14th coronal slice per animal for each group, starting from where the hippocampal formation is prominent (Bregma level 0.20 mm).

### 4.8. Neurobehavior

Two neurobehavioral assessments (open field and the negative geotaxis reflex) quantified behavioral responses 24 h after hypoxic exposure in P11/P12 pups from each of the experimental groups. Analysis of each test included the average of three trials per pup. To eliminate time of day differences in behavior, testing always occurred within the same 3-h block in the morning. A heating pad set at 37 °C prevented loss of body heat and pups rested between trials on the heating pad. The time each pup was separated from its dam did not exceed 20 min. Wiping down all surfaces of the behavioral apparatus with 70% ethanol between trials helped remove pup scent and reduce bacterial transfer.

Observations of exploratory activity in the open-field test enabled quantification of the number of times a rat’s head (specifically, the point between the ears) moved into a new 2 cm by 2 cm square in an enclosed, well-lit acrylic arena (38 cm high × 40 cm wide × 40 cm deep) during a 45-s interval. The negative geotaxis reflex tested the amount of time, up to 60 s, required for a pup placed head downward on a 25° mesh incline to turn 180° (indicated by position of its head and front paws).

### 4.9. Statistics

In all cases, to the number of animals studied. One-way ANOVA analyses with post-hoc comparisons using Fisher’s Paired Least Significant Difference tested significance among all endpoints, unless noted otherwise. For analysis of passive diameters, one-way ANOVA with repeated measures revealed significant differences. A D’Agostino–Pearson K2 test confirmed normal distributions of all data sets, and within ANOVA analyses, Levene’s test of equality verified homogeneity of variance. Violation of Levene’s led to the utilization of a Games–Howell post-hoc analysis.

In addition to assessing the effects of HI and MET between MUN groups, this study also examined the isolated effects of the MUN diet alone. The experimental design and group allocations within this study mirror those in a study published by our group, which examined gestational MET treatment and neonatal HI in ad libitum fed Control Diet (CD) rats [35]. The identical experimental design allowed for normalization of the MUN data presented herein relative to previously published CD data for each experimental endpoint. CD values equaled 100%, and normalized MUN values significantly greater or less than 100% indicated significant (*p* < 0.05) differences. Upward arrows on the figures indicate values that were significant greater in MUN-Sham than in CD-Sham endpoints, and conversely, downward arrows indicate values that were less in MUN-Sham than in CD-Sham endpoints. Statistical comparisons between MUN and CD values are shown by up and down arrows only for the Sham groups to simplify the graphical presentation of the analysis. The effects of HI and MET within the normalized MUN groups also used one-way ANOVA to determine significance as described above. Comparisons between MUN pup weights and CD pups at P11 used the Behrens–Fisher analyses to determine significance.

For each endpoint (Figure 2, Figure 4, Figure 5, Figure 6, Figure 8 and Figure 9), figure layouts included a set of top panels, which depicted raw MUN values and corresponding between-group comparisons, and a set of bottom panels, which depicted MUN values normalized relative to CD values and corresponding between-group comparisons. The raw MUN effects (top panels) represented the aggregate responses of MUN pups, whereas the normalized MUN effects (bottom panels) represented the isolated responses of MUN only. A significant comparison that existed in both raw and normalized responses denoted a strong effect of MUN. A significant comparison that existed only in the normalized responses signified opposing effects between the MUN and CD groups. Although the results presented do not explicitly indicate the previously reported effects of treatments in the CD groups [35], a significant effect that existed only in the raw responses represented a fundamental effect in the CD groups. Statistical significance implies *p* < 0.05. For each groupwise comparison, for the sake of visual clarity not all significant results appear in the figures, Results, or Discussion.

## 5. Conclusions

In summary, the present study demonstrated that under baseline Sham conditions, MUN programming during the latter half of gestation compromised neonatal behavior (Figure 10C,D), possibly secondary to vasogenic cerebral edema (Figure 8B) and mild alterations of region-specific cell swelling (Figure 9B). Behavioral changes associated with MUN programming also included reduced myogenic reactivity in pup middle cerebral arteries through depression of myofilament calcium sensitivity (Figure 6J,L), which may have compromised flow-metabolism coupling in brain areas involved in sensorimotor function. MUN programming also increased contraction-induced wall calcium mobilization (Figure 6K), which may have contributed to accelerated contractile differentiation of cerebrovascular vascular smooth muscle (Figure 4B,D). As revealed by the effects of MET during MUN, gestational corticosteroids: (1) contributed little to the effects of MUN on neurobehavior (Figure 10C,D); (2) promoted MUN-induced formation of cerebral edema (Figure 8B) and heterogeneous alterations of cell volume (Figure 9B); and (3) for the effects of MUN on cerebral arteries, corticosteroids enhanced myogenic calcium mobilization (Figure 6K) and limited contractile differentiation (Figure 4B,D). The greater values of circulating levels of corticosteroids, which occurred in MUN pups independent of gestational MET (Figure 2F), may also explain some effects of maternal undernutrition, given the widespread influences of corticosteroids on cerebral and cerebrovascular targets.

In addition to its effects on baseline cerebral and cerebrovascular characteristics, MUN also significantly modulated neonatal responses to mild hypoxic ischemia. HI had no significant effect on negative geotaxis in MUN pups, but unmasked an HI-induced improvement in open-field locomotion (Figure 10C,D), possibly through attenuation of cerebral edema formation in the ventral cortices (Figure 8B). In pup middle cerebral arteries, MUN programming enhanced HI-induced depression of calcium mobilization (Figure 6K,E), and produced greater levels of contractile differentiation (Figure 4B,D), which together could potentially reduce local cerebral perfusion and edema formation. As revealed by the effects of MET during MUN, gestational corticosteroids: (1) enhanced HI-induced exploration in the open-field test (Figure 10C,D), possibly through parallel attenuation of edema formation in the ventral cortex (Figure 8B); (2) contributed to HI-induced attenuation of contractility through limitation of increases in depolarization-induced calcium mobilization and myofilament sensitivity (Figure 6D–F); and (3) augmented HI-induced contractile differentiation (Figure 4B,D). Circulating levels of corticosterone in MUN pups were significantly greater than CD levels at 2 h but not 24 h post-HI (Figure 2E,F), implying that changes in circulating corticosterone levels at 24 h likely had no effect on HI-induced effects in other cells, tissues, or the brain. The possible influences of transient increases in corticosterone observed at 2 h post-hypoxia, as well as other corticosteroids, however, cannot be excluded. Interestingly, MUN programming had positive effects following mild HI on edema resolution and exploratory behavior, suggesting positive adaptative responses that might facilitate development of new approaches to better manage perinatal cerebrovasculature diseases.

In light of growing global patterns of food insecurity [2], the present study emphasizes that infants born from undernourished mothers may experience greater risk for developing neonatal cerebral edema and sensorimotor impairments possibly through programmed changes in neonatal cerebrovascular function. In clinical settings, these problems may be exacerbated by administration of synthetic glucocorticoids, which can be resistant to degradation by 11β-HSD2 [89]. Whereas the current findings in neonatal rats are of uncertain relevance to the effects of MUN in human infants, numerous studies suggest that the sequences and patterns of developmental events, particularly in relation to the role of developmental programming, are quite similar in the two species [90,91,92]. If so, the present findings emphasize that maintenance of appropriate levels of gestational corticosteroids throughout pregnancy should help minimize cerebral edema and support cerebrovascular development and maturation.

## Data Availability

The data presented in this study are available on request from the corresponding author.
